# Incidence and course of depression in multiple sclerosis in the multinational BEYOND trial

**DOI:** 10.1007/s00415-016-8146-8

**Published:** 2016-05-13

**Authors:** Sven Schippling, Paul O’Connor, Volker Knappertz, Christoph Pohl, Timon Bogumil, Gustavo Suarez, Stuart Cook, Massimo Filippi, Hans-Peter Hartung, Giancarlo Comi, Douglas R. Jeffery, Ludwig Kappos, Douglas S. Goodin, Barry Arnason

**Affiliations:** Department of Neuroimmunology and Multiple Sclerosis Research, Department of Neurology, University Medical Center Zurich, Frauenklinikstrasse 26, 8091 Zurich, Switzerland; St. Michael’s Hospital, Toronto, Canada; Teva Pharmaceuticals, Frazer, PA USA; Department of Neurology, Medical Faculty, Heinrich-Heine-Universität, Düsseldorf, Germany; Bayer HealthCare AG/Bayer Pharma AG, Berlin, Germany; University Hospital Bonn, Bonn, Germany; Bayer HealthCare Pharmaceuticals, Whippany, NJ USA; Rutgers-New Jersey Medical School, Newark, NJ USA; Ospedale San Raffaele, Milan, Italy; Vita-Salute University, Milan, Italy; Piedmont Health Care, Mooresville, NC USA; Neurology, Departments of Medicine, Clinical Research, Biomedicine and Biomedical Engineering, University Hospital Basel, Petersgraben 4, 4031 Basel, Switzerland; University of California at San Francisco, San Francisco, CA USA; Department of Neurology, University of Chicago, Chicago, IL USA

**Keywords:** Multiple sclerosis, Depression, Clinical trial, Interferon beta, Glatiramer acetate, BEYOND study

## Abstract

Early experience in MS generated concerns that interferon beta treatment might provoke onset or worsening of depression. The objective of the study was to compare depression incidence in relapsing–remitting MS patients receiving interferon beta-1b (IFNB-1b) or glatiramer acetate (GA) in the BEYOND trial. 891/897 (99 %) of English, French, Spanish and Italian speakers among 2244 patients randomized (2:2:1) to receive either IFNB-1b 500 µg, 250 µg, or GA 20 mg QD for 2–3.5 years submitted Beck Depression Inventory Second Edition (BDI-II) scores at screening and serially thereafter, in which scores ≥14 indicated depression. Baseline BDI-II scores ≥14 were reported in 232/891 patients (26.3 %), with no meaningful difference among the three treatment arms noted at this or at any other time during the study including trial end. Percentages of patients depressed by BDI-II scores deviated little in any arm at any time (IFNB-1b 500 µg: 24.7 %, IFNB-1b 250 µg: 24.4 %, GA: 32.4 %). Antidepressant usage was likewise similar among the three treatment arms (IFNB-1b 500 µg: 33.7 %, IFNB-1b 250 µg: 31.8 %, GA: 28.8 %) as was depression severity and the frequency with which non-blinded treating physicians recorded depression as an adverse event (IFNB-1b 500 µg: 17.2 %, IFNB-1b 250 µg: 17.0 %, GA: 14.4 %). Treating physicians attributed depression to IFNB-1b 250 µg therapy in 53.6 % and to GA in 21.9 % of instances. This large, prospective, randomized-controlled MS dataset showed no increased risk of depression above baseline values with standard or double-dose IFNB-1b or GA QD treatment.

**Electronic supplementary material:**

The online version of this article (doi:10.1007/s00415-016-8146-8) contains supplementary material, which is available to authorized users.

## Introduction

Multiple sclerosis is an inflammatory, demyelinating disease of the central nervous system that follows an unpredictable course [1]. Multifocal involvement of CNS white and gray matter can produce a multitude of clinical signs and symptoms that can cause accumulating physical impairment and disability. MS is frequently accompanied by cognitive and psychological changes that often lessen patient quality of life, occupational competence, and social interactions [2, 3]. Notably, depression is present at any given point in time in approximately one-quarter of MS patients versus 5 % of the population at large. Furthermore, the lifetime incidence of significant depressive symptomatology in MS patients can exceed 50 %, considerably higher than the 16 % estimated lifetime incidence of major depressive disorder in the US population at large [3–13]. Overall, it is now widely accepted that the rate of suicide in MS is significantly increased as compared to the underlying population [14].

Several interferon beta (IFNB) formulations are used to treat relapsing–remitting MS (RRMS) [6, 15]. Early experience with IFNB was interpreted by regulatory authorities as suggesting agent-related development and worsening of depressive symptoms. When depression develops in IFNB-treated patients, a careful consideration of treatment cessation has been advised [16–19]. In several reports, an association between IFNB therapy and depression has been proposed: included are case studies of individuals suffering from depression after starting IFNB treatment [20], depression recorded as adverse events during clinical trials and depression, accepted as such, based on response to non-validated questionnaires [21]. Yet, depression data collected during clinical trials in MS using validated instruments has consistently failed to document any such association [22].

Perceived links between IFNB and depression may be colored in part by the frequency of underlying depression in the absence of therapy; potential relationships between the distress that often follows a recent diagnosis of MS; dread of injections; the tendency for MS to worsen; and mood alterations triggered by concomitantly administered medications [23]. Depression is a well-established risk factor for suicide by MS patients [2, 24], such that a clear understanding of any association—or its absence—between IFNB therapies and depression is desirable. To this end, we retrospectively compared the incidence and severity of depression in 891 English-, French-, Spanish- or Italian-speaking patients among the 2244 patients enrolled in the Betaseron Efficacy Yielding Outcomes of a New Dose (BEYOND) trial.

## Patients and methods

Patients with RRMS were randomized in the BEYOND trial (NCT00099502) to receive either IFNB-1b 250 or 500 µg every other day or glatiramer acetate (GA) 20 mg daily in a 2:2:1 ratio using block randomization with regional stratification [25]. Patients were followed per protocol for 2 years, with patients continuing on randomized therapy until the last entered patient had reached the 2-year study end. Patients randomized early during recruitment were followed for a maximum of up to 3.5 years. Treating physicians and patients were aware of allocation to IFNB or GA, but were blinded to IFNB dose. Evaluating physicians were blinded to all treatment assignments [25]. BEYOND was performed in accordance with Good Clinical Practice and the International Conference on Harmonization (ICH) guidelines. The institutional review boards of all participating centers approved the study protocol. Patients provided written informed consent.

### Study population

Treatment-naïve patients with RRMS from 26 countries who met the 2001 McDonald diagnostic criteria for MS [26] were eligible for inclusion in BEYOND if aged between 18 and 55 years, had at least one relapse in the year before study entry, and had a baseline Expanded Disability Status Scale (EDSS) [27] score of ≤5. Patients were excluded from the study if they presented with progressive forms of MS, or if they had received either experimental or approved treatment for MS. Importantly, patients with a history of severe depression, a previous suicide attempt, or current suicidal ideation were also excluded.

### Assessment of depression

Depression and suicidal ideation were assessed on a quarterly basis (every 12 weeks) using the Beck Depression Inventory Second Edition (BDI-II) [28] in English-, French-, Spanish- and Italian-speaking patients, because validated versions were only available in these languages. The BDI-II is a self-administered rating inventory consisting of 21 items that measures attitudes and symptoms suggestive of depression, including feelings of hopelessness and guilt, irritability and fatigue, as well as sleep patterns and loss of appetite. Each item is rated on an intensity scale of 0–3, with a maximum score of 63. Total scores of ≤13 indicate no or insignificant depression, while scores in the range of 14–19 suggest mild; 20–28, moderate; and ≥29, severe depression [28].

Guided by the Goldman Consensus statement recommendations on scoring depression in MS, BDI-II scores were dichotomized: ≤13 taken as no or minimal depression and ≥14 as presence of depression—be it mild, moderate, or severe [29].

Treating physicians, as part of their routine monitoring of adverse events, recorded their subjective estimation of depression occurrence at each office visit and provided an opinion as to whether there was a relationship between treatment given and depression in patients thought to be depressed.

### Antidepressant usage

Information on antidepressant use by study participants was collected at each office visit. The co-medication database was searched using the following search terms: “antidepressants,” “nonselective monoamine reuptake inhibitors,” “selective serotonin reuptake inhibitors,” “nonselective monoamine oxidase inhibitors,” “monoamine oxidase A inhibitors,” “other antidepressants,” “antidepressants in combination with psycholeptics,” and “lithium.”

### Statistics

Descriptive statistics and Fisher’s exact test at the 0.05 level of significance (post hoc) were used for data analyses.

## Results

### Study population

Altogether, 2244 patients were randomized in the multinational BEYOND study. The majority were female (1562/2244; 69.6 %) and Caucasian (2045/2242; 91.2 %). Most patients (1786/2244; 79.6 %) had an EDSS score at screening of 1.0–3.5. The mean age at disease onset was 31.0 ± 9.1 years and mean disease duration was 5.3 ± 5.8 years (Table 1). A total of 24 patients did not receive treatment with study medication and accordingly were omitted from the per-protocol analysis. Baseline demographics and disease characteristics were similar across the treatment groups, as described in more detail elsewhere [25].

**Table 1:** Baseline characteristics of participants randomized in the BEYOND study

	IFNB-1b 500 µg	IFNB-1b 250 µg	GA	Total
	*n* = 899	*n* = 897	*n* = 448	*N* = 2244
Duration of disease				
*N*	899	896	448	2243
Mean (range, SD), years	5.4 (0–33, 5.8)	5.3 (0–42, 5.8)	5.1 (0–29, 5.6)	5.3 (0–42, 5.8)
Age at onset				
*N*	899	896	448	2243
Mean (range, SD), y	31.1 (9–55, 9.2)	31.1 (10–53, 9.2)	30.6 (8–55, 9.0)	31.0 (8–55, 9.1)
Number of relapses during the previous year				
*N*	899	896	448	2243
Mean (range, SD),	1.6 (0–8, 0.8)	1.6 (1–5, 0.7)	1.6 (0–5, 0.8)	1.6 (0–8, 0.7)
Total number of previous relapses				
*N*	894	888	448	2230
Mean (range, SD)	3.5 (1–30, 2.6)	3.5 (1–30, 2.7)	3.7 (1–21, 2.6)	3.5 (1–30, 2.6)

*BEYOND* Betaseron Efficacy Yielding Outcomes of a New Dose [trial], *GA* glatiramer acetate, *IFNB-1b* interferon beta-1b

### Depression assessed by BDI-II

The BDI-II enabled assessment of patients from Australia, Canada, the USA, France, Italy, and Spain. Validated language versions were not available for 60.0 % (1347/2244) of randomized patients. A total of 39.7 % of randomized patients (891/2244) had BDI-II scores recorded at screening (proportion in each treatment arm: IFNB-1b 500 µg, 40.2 % [361/899]; IFNB-1b 250 µg, 39.8 % [357/897]; GA, 38.6 % [173/448]). Only six patients eligible for BDI-II testing did not perform a screening assessment. Of patients with screening BDI-II scores, 74.0 % overall (659/891) had BDI-II scores ≤13, indicating no or minimal depression, while 26.0 % (232/891) had scores ≥14, suggesting mild to severe depression (proportion in each treatment arm: IFNB-1b 500 µg, 24.7 % [89/361]; IFNB-1b 250 µg, 24.4 % [87/357]; GA, 32.4 % [56/173]). No significant differences in depression severity were found between treatment arms at screening (IFNB-1b 500 µg vs. GA, *p* = 0.062; IFNB-1b 250 µg vs. GA, *p* = 0.060).

Patients with BDI-II scores ≥14 had higher baseline EDSS scores (*p* < 0.0001) and more relapses in the 2 years prior to enrollment (*p* = 0.0017) than patients with scores ≤13 (Table 2). However, patients with higher baseline depression scores had fewer gadolinium-enhancing lesions and lower gadolinium-enhancing lesion volume at baseline (*p* = 0.0134 and *p* = 0.0163, respectively) than participants with screening BDI-II scores ≤ 13.

**Table 2:** Baseline characteristics of patients by BDI-II scores at screening

	Screening BDI-II score ≤ 13	Screening BDI-II score ≥ 14
	*n* = 659	*n* = 232
Female		
*n*/*N*	479/659 (72.7 %)	181/232 (78.0 %)
*p* value		0.1175
Caucasian		
*n*/*N*	577/658 (87.7 %)	195/231 (84.4 %)
*p* value		0.2141
Age		
*N*	659	232
Mean (range, SD), years	38.0 (18–57, 9.1)	38.0 (18–55, 9.0)
*p* value		0.8577
Duration of disease		
*N*	659	232
Mean (range, SD), years	4.7 (0–42, 5.8)	4.9 (0–32, 5.7)
*p* value		0.4159
Relapse rate in the previous 2 years		
*N*	657	232
Mean (range, SD)	1.9 (1.0–10.0, 1.0)	2.2 (1.0–15.0, 1.3)
*p* value		0.0017
Two or more relapses in the previous 2 years		
*n*/*N*	433/657 (65.9 %)	173/232 (74.6 %)
*p* value		0.0173
EDSS		
*N*	651	227
Mean (range, SD)	2.0 (0–5.5, 1.1)	2.7 (0–6.0, 1.2)
*p* value		<0.0001
T2 lesion volume (cm^3^)		
*N*	657	230
Mean (range, SD)	7.0 (0.1–72.7, 8.4)	6.6 (0.1–102.0, 9.6)
*p* value		0.2312
T1-hypointense lesion volume (cm^3^)		
*N*	648	227
Mean (range, SD)	1.2 (0–26.1, 2.5)	1.1 (0–39.1, 2.9)
*p* value		0.3305
One or more Gd-enhancing lesions		
*n*/*N*	295/659 (44.8 %)	82/232 (35.3 %)
*p* value		0.0134
Gd-enhancing lesion volume (cm^3^)		
*N*	651	229
Mean (range, SD)	0.22 (0–6.7, 0.6)	0.16 (0–5.8, 0.5)
*p* value		0.0163
Normalized brain volume (cm^3^)		
*N*	648	228
Mean (range, SD)	1502.9 (1171–1804, 106.7)	1494.7 (1142–1764, 103.2)
*p* value		0.3121

*BDI-II* Beck Depression Inventory Second Edition, *EDSS* Expanded Disability Status Scale, *GD* gadolinium

**Fig. 1:**
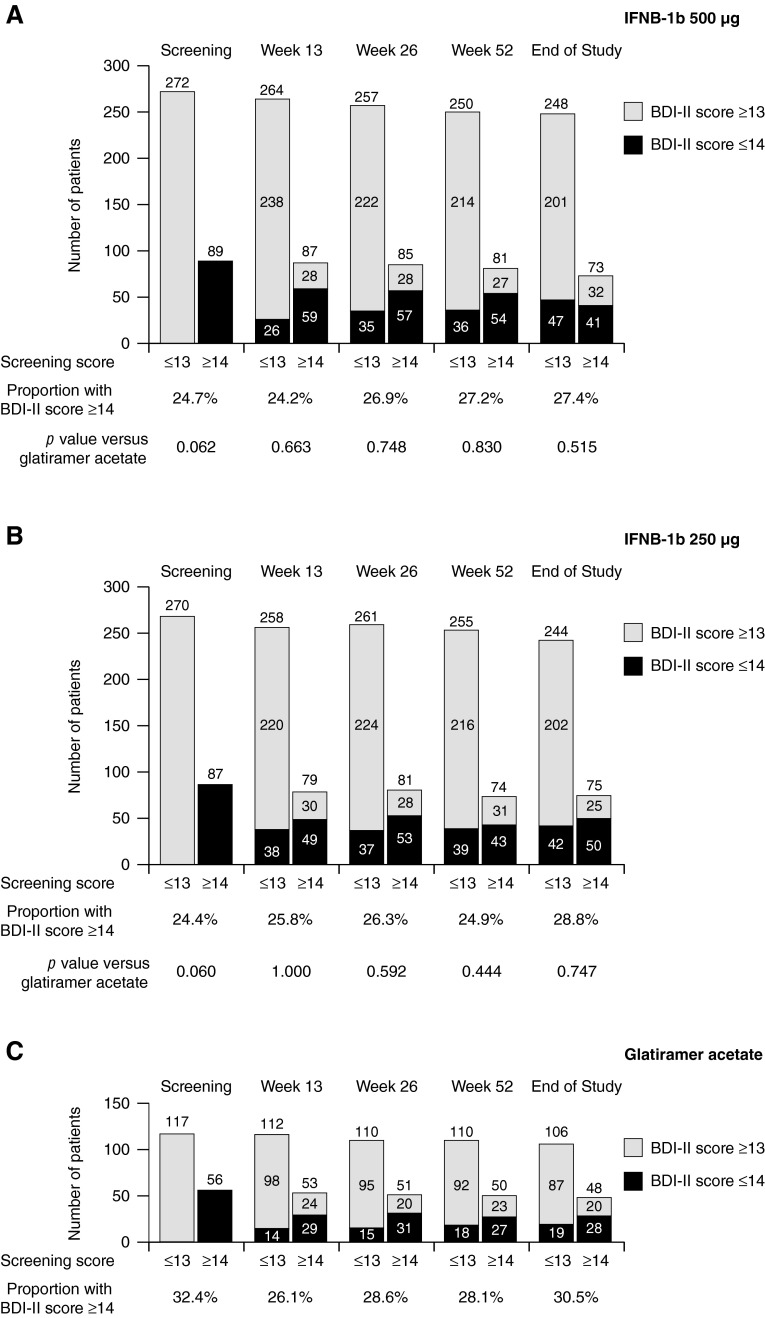
Number of patients with BDI-II scores at selected time points stratified by BDI-II score at screening and treatment assignment. Fisher’s exact test was used for comparison

The number and relative proportion of patients in each treatment group with BDI-II scores ≤13 or ≥14 at each time point stratified by BDI-II score at screening are presented in Fig. 1 and Supplemental Fig. 1. At all time points, the proportion of patients with depression was marginally smaller for both IFNB-1b doses than for GA, but the differences were not significant (IFNB-1b 500 µg vs. GA, *p* = 0.882; IFNB-1b 250 µg vs. GA, *p* = 0.879).

**Fig. 2:**
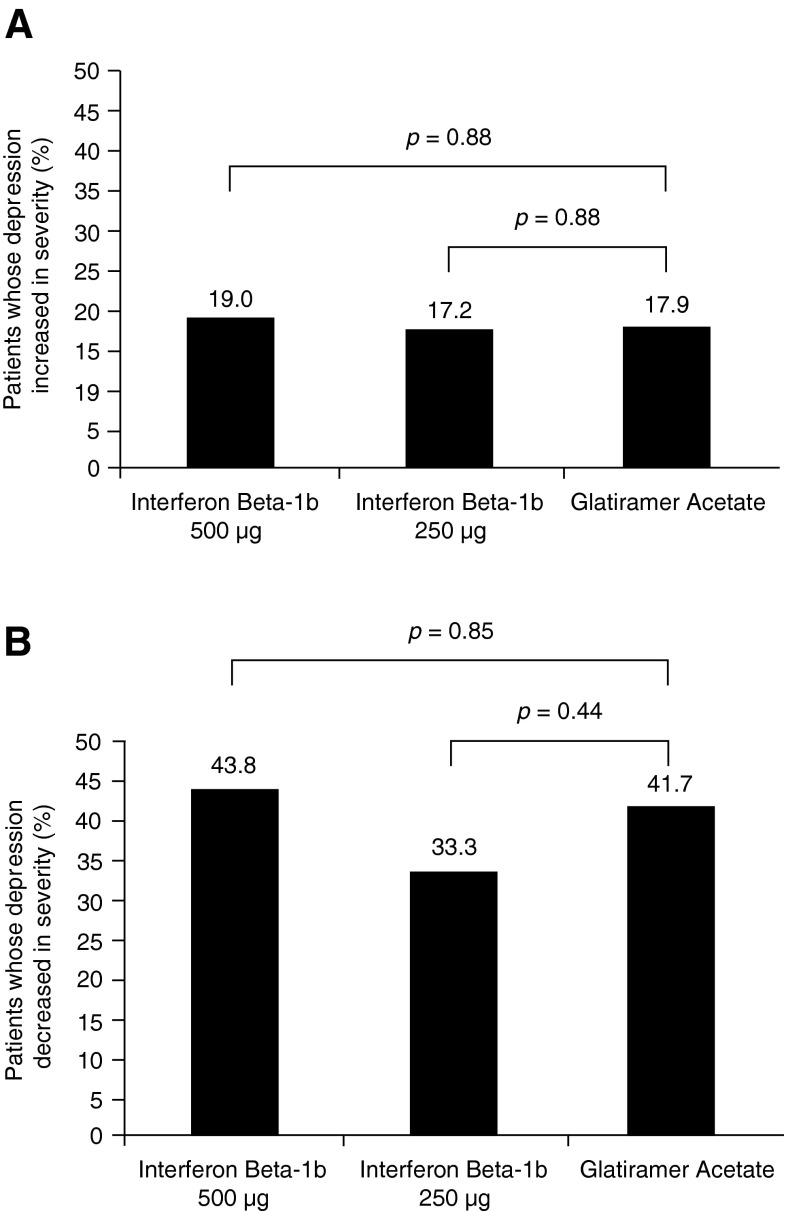
Proportion of patients with **a** BDI scores ≤13 at screening who had BDI-II scores of ≥14 at the end of the study (*n* = 794); **b** BDI-II scores ≥14 at screening who had BDI-II scores ≤13 at the end of the study (*n* = 794). Fisher’s exact test was used for comparison

At study end, BDI-II scores were provided by 794 patients, 567 of whom (71.4 %) were not depressed (BDI-II score ≤13). Of 598 patients not depressed at baseline (scores ≤13), 108 (18.1 %) became depressed at some time during the trial (scores rose to ≥14). The upward shift was similar among the three treatment arms (IFNB-1b 500 µg, 47/248 [18.9 %]; IFNB-1b 250 µg, 42/244 [17.2 %]; GA, 19/106 [17.9 %]; IFNB-1b 500 µg vs. GA, *p* = 0.88; IFNB-1b 250 µg vs. GA, *p* = 0.88; Fig. 2a).

In contrast, among the 196 patients who were depressed at baseline (BDI-II score ≥14), depression lifted (BDI-II score fell to ≤13) during the trial in 77 (39.3 %), with once again an indistinguishable downward shift among treatment arms (IFN-1b 500 µg, 32/73 [43.8 %]; IFN-1b 250 µg, 25/75 [33.3 %]; GA, 20/48 [41.7 %]; IFN-1b 500 µg vs. GA, *p* = 0.85; IFN-1b 250 µg vs. GA, *p* = 0.44; Fig. 2b).

### Depression as a clinically determined adverse event

**Fig. 3:**
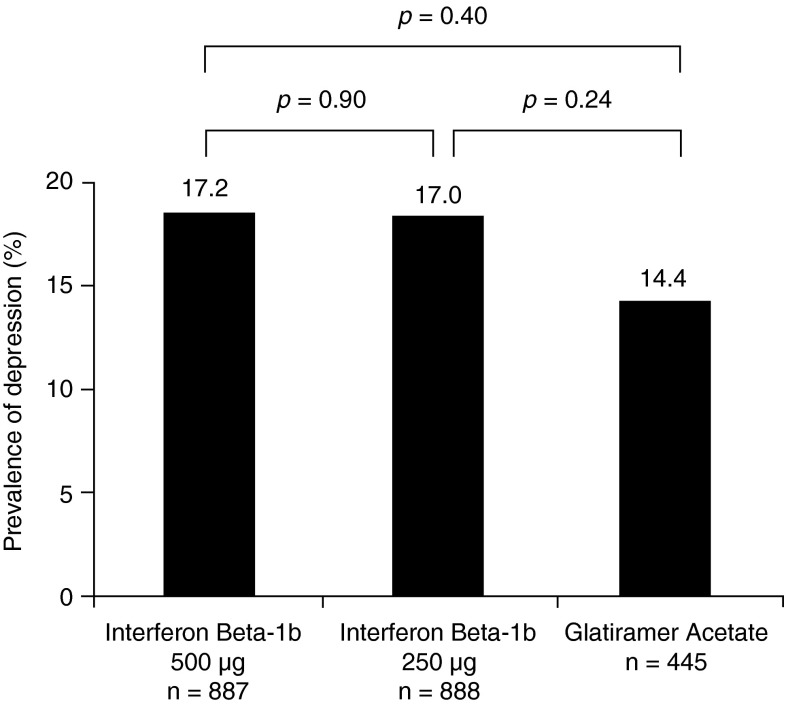
Proportion of patients in whom depression was reported as an adverse event at any given time during the study (*n* = 2220). Fisher’s exact test used for comparison

The frequency with which depression was reported as an adverse event by non-blinded treating physicians was similar for IFNB-1b 500 µg (153/887, 17.2 %); IFNB-1b 250 µg (151/888 17.0 %); and GA (64/445 14.4 %)—IFNB-1b 500 µg vs. GA (*p* = 0.40); IFNB-1b 250 µg vs. GA (*p* = 0.24) (Fig. 3).

Treating physicians viewed depression severity as mild to moderate in most cases (IFNB-1b 500 µg, 137/153 [89.5 %]; IFNB-1b 250 µg, 137/151 [90.7 %]; GA, 61/64 [95.3 %]). Depression as the basis for premature medication discontinuation was recorded in <1 % of patients (IFNB-1b 500 µg, 8/887 [0.9 %]; IFNB-1b 250 µg, 8/888 [0.9 %]; GA, 0/445 [0 %]).

### Depression ascribed to treatment

Although reporting of depression as an adverse event was similar across the three treatment arms, treating physicians viewed depression as therapy related more than twice as often for patients treated with IFNB-1b as for those treated with GA (IFNB-1b 500 µg, 85/153 [55.6 %]; IFNB-1b 250 µg, 81/151 [53.6 %]; GA, 14/64 [21.9 %]). Of note, treating physicians knew which patients had been randomized to IFNB-1b (although dose was not known) or to GA.

### Incidence of suicidal ideation and suicide

The incidence of suicidal ideation in the BEYOND study was low in all three treatment groups (IFNB-1b 500 µg, 2/887 [0.2 %]; IFNB-1b 250 µg, 3/888 [0.3 %]; GA, 1/445 [0.2 %]). Nevertheless, suicidal ideation—though rare—was attributed by the treating physician to IFNB-1b treatment in 5/5 cases, but not to GA in the single recorded instance of suicidal ideation in this treatment arm.

Two patients, both randomized to IFNB-1b 250 µg (2/888, 0.2 %), attempted suicide at 0.7 and 2.8 years into the study. One attempt was thought to possibly be attributable to treatment. Two additional patients, one randomized to IFNB-1b 500 µg (1/887, 0.1 %) and one to GA (1/445, 0.2 %), completed suicide at 1.8 and 0.6 years into the study, respectively. Neither instance was deemed to be related to treatment.

### Antidepressant usage

The proportions of patients treated with antidepressant medication at some point during the BEYOND study were similar across the treatment arms: IFNB-1b 500 µg, 33.7 % (299/887); IFNB-1b 250 µg, 31.8 % (282/888); GA, 28.8 % (128/445) (IFNB-1b 500 µg vs. GA, *p* = 0.07; IFNB-1b 250 µg vs. GA, *p* = 0.28).

## Discussion

The incidence and severity of depression in RRMS patients treated with either of two doses of IFNB-1b or with GA in the multinational BEYOND clinical trial were evaluated prospectively using a validated instrument for assessing depression. The exclusion of patients with a clinical history of severe depression, attempted suicide or suicidal ideation reduced the potential confounders for this analysis. Twenty-six percent of entrants had a BDI-II score ≥14 at screening, indicative of depression of at least mild severity. Patients with BDI-II scores ≥14 at screening had higher relapse rates in the 2 years preceding entry and higher EDSS scores at baseline than patients with BDI-II scores <13. Intriguingly, they also had fewer gadolinium-enhancing lesions, suggesting that clinically apparent relapses might be a more reliable indicator of depression than gadolinium positivity.

At trial entry, numerically fewer patients randomly assigned to IFNB-1b (either dose) had BDI-II total scores indicative of clinical depression than patients assigned to GA. These differences, while minor, persisted over the course of the trial, although they never reached significance at any time point. More importantly, similar proportions of patients in all three arms without depression at study entry progressed to BDI-II scores indicative of depression during the trial, while comparative numbers of patients in all three arms with BDI-II score suggestive of depression at study entry reverted to non-depressed status during the trial. These two trends largely offset one another, such that the incidence of depression remained relatively constant among all three arms throughout the course of the trial. Antidepressant usage was likewise comparable across all three treatment arms.

Despite the above, treating physicians were far more likely to cite depression as a complication of IFNB-1b treatment than as a complication of GA treatment. Labels for IFNB warn of depression and suicide, whereas labels for GA carry no such warning; this difference may have accounted for an attribution bias. Although the non-blinding of physicians could be considered a limitation of the study, the frequency with which non-blinded treating physicians clinically stated depression as an adverse event was substantially lower than the frequency with which depression was suggested by the BDI-II across all three treatment groups. Thus, the data presented here suggest that awareness of the patient’s treatment did not substantially influence reporting of clinical depression and underscore the value of screening with a validated instrument to get a full sense of depression frequency in MS.

Another possible limitation of the present study is the absence of a placebo-treated control group. Consequently, the issue of whether IFNB-1b and GA therapies are both associated with depression, or neither therapy is so associated, cannot be resolved definitively by the available data presented here. In addition, only a relatively small percentage of patients (approximately, 40 %) completed the BDI. However, it is important to note that the percentage of patients with BDI data in each treatment group was similar. Nonetheless, the present study provides strong support to a considerable body of literature favoring the view that neither agent is linked to aggravating or inducing clinical depression. First, GA has not been perceived to be associated with depression in placebo-controlled or open-label studies nor as a spontaneously reported adverse event. Second, outcomes from the BEYOND study match data generated in other randomized, placebo-controlled trials of IFNB: no significant difference in the occurrence of patient-reported depression was found between IFNB-1b and placebo in the original IFNB-1b trial [30]. Similarly, in the 2-year study comparing intramuscular IFNB-1a with placebo, BDI-II scores did not differ at any time point between treatment groups [6]. In the “Prevention of Relapses and Disability by IFNB-1a Subcutaneously in Multiple Sclerosis” (PRISMS) study, no relationship between IFNB-1a treatment and clinically significant depression was found over 24 months (relative risk, 0.3–1.8) compared with placebo [31]. Furthermore, in a pooled analysis of 6 controlled studies and 17 non-controlled trials, subcutaneous IFNB-1a therapy was not linked to an increase in depression severity or suicide risk [22] and a controlled, epidemiologic study that assessed depression in 50 Italian RRMS patients and 50 healthy controls using a variety of methods (Structured Clinical Interview for Diagnostic and Statistical Manual of Mental Disorders (DSM-IV), BDI, and State Trait Anxiety Inventory) concluded that IFNB therapy was not a risk factor for depression [32]. A sizable head-to-head (*n* = 764) clinical trial comparing subcutaneous IFNB-1a versus GA found no significant differences in the proportions of patients reporting depression as an adverse event [33]. Last, IFNB-1b- and GA-treated patients identified in a health-care database exhibited no differences in antidepressant usage, a useful proxy for depression incidence [34]. Collectively, these data—reinforced by the results presented here—provide compelling evidence to support the hypothesis that IFNB neither causes nor exacerbates depression in patients with MS.

We recognize that MS patients with a history of depression may be more vulnerable to subsequent depression than those without such a history, and that disease activity may herald future depression. In the BEYOND study, patients with active depression or a clinical history of severe depression were excluded, and the majority of participants exhibited low disease activity over the 3-year study duration. While the absence of patients with a previous history of depression in the BEYOND study population may limit the generalizability of our findings, no biological signal was detected in this patient cohort and IFNB-1b was not found to induce or exacerbate depressive symptoms.

## Electronic supplementary material

Below is the link to the electronic supplementary material.

Supplementary material 1 (DOCX 618 kb)
