# Supplementary material for: Incidence and course of depression in multiple sclerosis in the multinational BEYOND trial
Source: J Neurol. 2016 May 13;263:1418–26. doi: 10.1007/s00415-016-8146-8 (PMC4929160; doi:10.1007/s00415-016-8146-8)
Supplement: Supplementary file 1 — Supplementary material 1 (DOCX 618 kb) [file 415_2016_8146_MOESM1_ESM.docx]

Incidence and course of depression in multiple sclerosis in the multinational BEYOND trial

Sven Schippling^1^, Paul O’Connor^2^, Christoph Pohl^3,4^, Rupert Sandbrink^3,5^, Timon Bogumil^3^, Francis Boateng^3^, Gustavo Suarez^6^, Stuart Cook^7^, Massimo Filippi^8^, Hans-Peter Hartung^5^, Giancarlo Comi^9^, Douglas R. Jeffery^10^, Ludwig Kappos^11^, Douglas S. Goodin^12^ and Barry Arnason^13^ on behalf of the BEYOND Study Group

^1^University Medical Center Zurich, Zurich, Switzerland

^2^St. Michael’s Hospital, Toronto, Canada

^3^Bayer HealthCare AG /Bayer Pharma AG, Berlin, Germany

^4^University Hospital Bonn, Bonn, Germany

^5^Heinrich-Heine-Universität, Düsseldorf, Germany

^6^Bayer HealthCare Pharmaceuticals, Whippany, New Jersey, US

^7^University of Medicine and Dentistry New Jersey Medical School, Newark, New Jersey, US

^8^Ospedale San Raffaele, Milan, Italy

^9^Vita-Salute University, Milan, Italy

^10^Wake Forest University Baptist Medical Center, Winston-Salem, North Carolina, US

^11^Neurology, Departments of Medicine, Clinical Research, Biomedicine and Biomedical Engineering, University Hospital Basel, Petersgraben 4, CH-4031, Basel, Switzerland

^12^University of California at San Francisco, San Francisco, California, US

^13^Department of Neurology, University of Chicago, Chicago, Illinois, US

### Corresponding author:

Sven Schippling, Department of Neuroimmunology and Multiple Sclerosis Research, Department of Neurology, University Medical Center Zurich, Frauenklinikstrasse 26, CH-8091 Zurich, Switzerland. Tel: +41 44 255 1218; Fax: +41 44 255 4507.

Email: sven.schippling@usz.ch

**Supplemental Figure 1:** Number of patients with BDI-II scores at each time point stratified by BDI-II score at screening and treatment assignment. Fisher’s exact test used for comparison.
